# Nearly Complete Response of Brain Metastases from HER2 Overexpressing Breast Cancer with Lapatinib and Capecitabine after Whole Brain Irradiation

**DOI:** 10.1155/2013/234391

**Published:** 2013-09-26

**Authors:** Esin Oktay, Özlem Yersal, Nezih Meydan, Mehmet Sağıroğlu, Ömer Uyanık, Sabri Barutca

**Affiliations:** Department of Medical Oncology, Faculty of Medicine, Adnan Menderes University, 09010 Aydın, Turkey

## Abstract

Trastuzumab treatment does not prevent intracranial seeding and is largely ineffective for established central nervous system metastasis in HER2 overexpressing breast cancer patients. Combination therapy of lapatinib and capecitabine may be an effective treatment option for brain metastasis of HER2-positive breast cancer. We report a patient with breast cancer overexpressing HER-2 where brain metastases were successfully treated with radiation and a combination of lapatinib and capecitabine.

## 1. Introduction

HER-2 overexpression is associated with poorer disease-free and overall survival in breast cancer patients and with a tendency for visceral site metastasis [1]. Despite the positive effect on response rate and overall survival, one third of the patients treated with trastuzumab develop brain metastasis. Trastuzumab is a highly selective monoclonal antibody which targets the extracellular domain of the HER2 receptor and does not fully cross the blood-brain barrier (BBB). 

Lapatinib is an oral small molecule tyrosine kinase inhibitor which targets the C-terminus domain of both the HER2 and EGFR receptors. Due to its small size, it can be across the BBB more effective than the larger trastuzumab molecule [2]. Here, we report a patient with HER-2 overexpressing breast cancer where brain metastases were successfully treated with lapatinib and capecitabine combination regimen.

## 2. Case Presentation

A forty-three-years old female patient was admitted to our hospital due to the right breast mass. Bilateral mammography revealed an ill-defined 15 mm mass lesion in the right breast. An invasive ductal carcinoma was diagnosed by core needle biopsy of the lesion and the tumor was estrogen receptor (ER)-negative, progesterone receptor (PgR)-negative, human epidermal growth factor receptor 2 (Her2)-positive (+++), and Ki-67 50%. After the modified radical mastectomy operation, the pathological examination revealed that an invasive tumor was 5 cm in diameter and that 39 out of 40 lymph nodes were metastatic.

She received four cycles of EC (90/600 mg/m^2^ doses, resp.) chemotherapy regimen followed by weekly paclitaxel and trastuzumab combination (paclitaxel 80 mg/m^2^; trastuzumab 2 mg/kg after the loading dose of 4 mg/kg) for 12 weeks. At the end of the paclitaxel treatment, trastuzumab was planned 6 mg/kg maintenance dose every 21 days for totally one year. 

**Figure 1:**
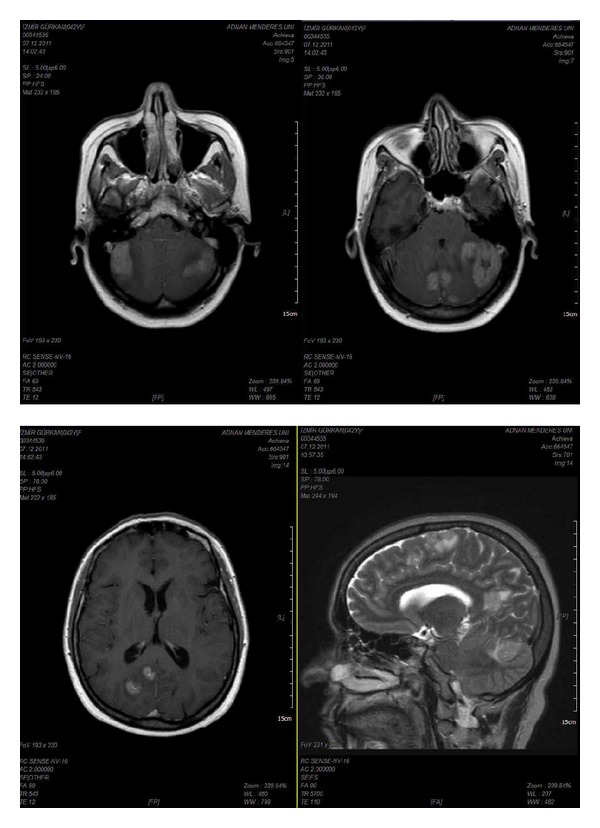
Magnetic resonance imaging scans demonstrating multiple brain lesions.

**Figure 2:**
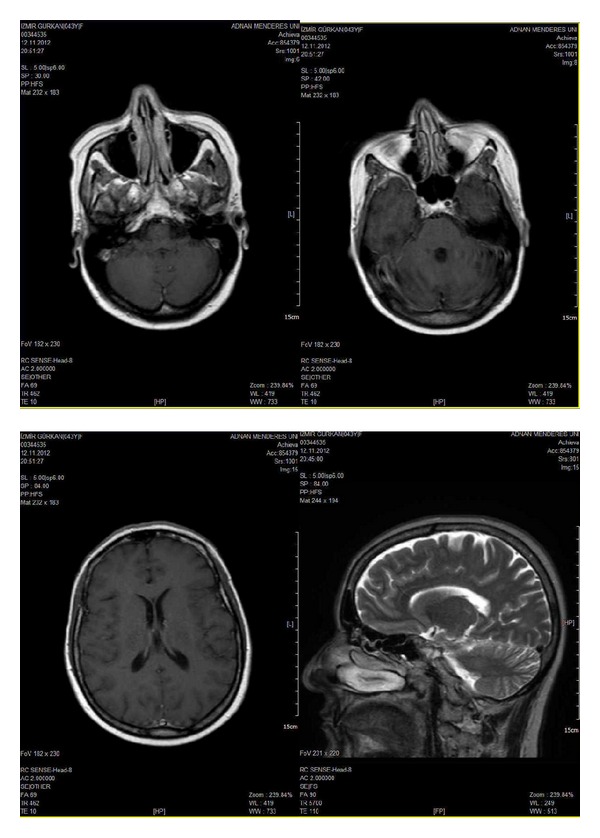
Magnetic resonance imaging scans demonstrating near complete resolution of brain metastases after lapatinib plus capecitabine treatment.

However, after the first course of the maintenance trastuzumab, she complained of dizziness and progressive imbalance. Magnetic resonance imaging (MRI) of the brain revealed multiple brain lesions in cerebellum, periventricular, and supraventricular white matter accompanied by prominent vasogenic edema (Figure 1). The patient received whole brain radiation therapy with a total dose of 3000 cGy which was administered in 10 fractions of 300 cGy. After the completion of radiotherapy, lapatinib and capecitabine combination (1250 mg/day and 1500 mg twice daily, 14/21 days, resp.) was started. Control MRI showed stable brain metastases one month later. A partial CNS response was documented with a decrease of about 30% in her brain metastases after two cycles of chemotherapy. Metastatic lesions kept regressing till sixth month of treatment and there were no masses except milimetric cerebellar metastases on MRI (Figure 2). They are stable at eighteen months of therapy while there are no other visseral metastases.

## 3. Discussion

Human epidermal growth factor receptor 2 (HER2/ErbB2) is a member of the ErbB family of tyrosine kinase receptors. Activation of HER2 regulates normal cell growth; dysregulated HER2 activation supports tumorigenesis via cell proliferation, migration, survival, and angiogenesis [3]. HER2-positive tumors occur in 25% of all breast cancers [1]. 

Overexpression of HER2 indicates poor prognostic factor for overall and disease-free survival, and it is a risk factor for the development of brain metastases. Gabos et al. followed up 301 Her2-positive and 363 Her2-negative breast cancer patients during 3.9 years. Brain metastasis was found to be 9% in Her2-positive cases and 1.9% in those with negative Her2 [4].

Several retrospective analyses of HER2 positive metastatic BC patients treated with trastuzumab-based therapies showed that about 30% of these patients develop BMs [2], and in more than 50% of these cases, BMs occur in patients with either responsive or stable disease at extracranial sites. This patient developed brain metastases under treatment with trastuzumab in the adjuvant setting.

Trastuzumab has limited penetration through the blood brain barrier. The ratio of trastuzumab level in serum to cerebrospinal fluid is found to be 430 : 1, thus making the brain vulnerable for the development of metastases [1]. Lapatinib is a small molecule and reversible inhibitor of both HER1 and HER2. Its molecular weight is very low (<1 kDa). It has theoretical ability to cross the BBB. Preclinically this has been demonstrated in mice with HER-2 overexpressing brain metastases. Treatment with lapatinib resulted in a significant decrease in tumor volume which was attributed to a decrease in HER-2 phosphorylation and cell proliferation [5]. A phase II study with single agent lapatinib 750 mg bid was undertaken in patients with HER2-positive CNS metastases who had shown progression in CNS lesions after whole-brain radiotherapy, and clinical and radiological responses were observed [6]. 

Most clinical or preclinical data showed that lapatinib and capacitabine combination therapy is more effective than lapatinib or capacitabine monotherapy in breast cancer with brain metastases. In a phase II trial, HER2 positive metastatic breast cancer patients who developed brain metastases after trastuzumab therapy were treated with lapatinib monotherapy. Seven % of patients had >50% reduction in brain metastasis volume, 6% had a partial response, and 42% had stable disease in more than 8 weeks [7]. Patients whose CNS disease was progresssed on lapatinib treatment, capecitabine and lapatinib was administered. Twenty % of patients had 50% reduction in tumor volume, 20% of patients had partial CNS response, 39% had stable disease in more than 8 weeks [8].

This case report describes the near complete resolution of brain metastases in a 43-year-old woman with HER2-positive metastatic breast cancer who was treated with a combination of lapatinib plus capecitabine. This result is in agreement with previous reports that this combination is more effective in treatment of brain metastases. In the LANDSCAPE trial, 45 patients with brain metastases from HER2+ breast cancer received lapatinib plus capecitabine prior to radiation therapy, reported a CNS objective response of 67%, with a median time to tumor progression of 5.5 months [9]. 

In randomized phase III trial (EGF100151) lapatinib plus capacitabine versus capacitabine for incidence of brain metastases as site of first progression, the combination of lapatinib plus capecitabine achieved a significant reduction in the incidence of brain metastases as site of first progression (2% versus 6%; *P* = 0.045) [10].

In the Lapatinib Expanded Acces Program (LEAP), 138 patients with progresive CNS metastases (patients received prior capacitabine therapy) were enrolled, and the CNS objective response rate was 18% with combined lapatinib plus capacitabine [11]. 

In some studies, other agents were used instead of capecitabine. A phase II study tested lapatinib plus capecitabine versus lapatinib plus topotecan in patients with HER2+ breast cancer with brain metastases. The primary end point was CNS objective response. No response was observed in lapatinib plus topotecan arm. The objective response rate in the lapatinib plus capacitabine arm was 38% [12]. 

In conclusion, trastuzumab treatment does not prevent intracranial seeding and is largely ineffective for established CNS disease. Today, women with HER2-positive BM are surviving longer. Most clinical studies show that LC is an active combination after the development of BMs and may further improve the prognosis of patients with BMs from HER2+ BC compared with the trastuzumab-based therapies. 

This patient had nearly complete response with this combination therapy. She has been alive without any complaint for eighteen months. There is no progresion in CNS lessions and there is no other visseral metastases. Based on our single case observation, capacitabine and lapatinib combination therapy seems as the best choice for breast cancer patients with only CNS metastases.

 On the other hand, prevention from BM is important too. Many risk factors are associated with the development of BM, such as young age, hormone receptor-negative primary tumors, HER2+ tumors, and heavy burden of disease (large primary tumors, lymph node involvement, prior lung, liver, or bone metastases, increased number of metastatic sites, and elevated lactate dehydrogenase [LDH] levels) [1]. In breast cancer patients with high risk of brain metastasis, whole-brain irradiation may be studied in controlled clinical trials.
